# Lifecycle of the invasive omnivore, *Forficula auricularia*, in Australian grain growing environments

**DOI:** 10.1002/ps.6206

**Published:** 2020-12-22

**Authors:** Matthew Binns, Ary A Hoffmann, Maarten van Helden, Thomas Heddle, Matthew P Hill, Sarina Macfadyen, Michael A Nash, Paul A Umina

**Affiliations:** ^1^ Commonwealth Scientific and Industrial Research Organisation Agriculture & Food Canberra Australia; ^2^ Bio21 Institute, School of Biosciences, University of Melbourne Melbourne Australia; ^3^ South Australian Research and Development Institute Adelaide Australia; ^4^ University of Adelaide Adelaide Australia; ^5^ Commonwealth Scientific and Industrial Research Organisation Data61 Canberra Australia; ^6^ School of Life Science, La Trobe University Melbourne Australia; ^7^ Cesar Australia Melbourne Australia

**Keywords:** European earwig, phenology, agriculture, grain crops, Australia

## Abstract

**BACKGROUND:**

The European earwig, *Forficula auricularia* (L.) (Dermaptera: Forficulidae), is regarded as an important beneficial in many orchard environments but has the potential to be a plant pest in other systems, including to grain crops. Due to its agricultural importance, the lifecycle of *F. auricularia* has been widely studied in North America and Europe. However, much less is known in the southern hemisphere, including Australia where *F. auricularia* has been present for over 170 years.

**RESULTS:**

To elucidate the lifecycle of *F. auricularia*, we monitored five sites in grain‐growing areas of southern Australia using two different trap types. Adults were found year‐round, but most prevalent from late‐spring to mid‐winter. First instars were typically found from mid to late winter. Second, third and fourth instars occurred from winter through to late spring. The seasonal development of *F. auricularia* in Australia may be much earlier than observed in comparable North American studies. Degree day modelling highlights variation in development times across the active season for *F. auricularia* across our sites.

**CONCLUSION:**

*Forficula auricularia* is well adapted to the Australian grain growing environments. The timing of egg hatching aligns closely with crop emergence, juveniles then develop alongside the crop, and adult development occurs by harvest time in late spring. These findings are important given that many of these crops (canola, lucerne, pulses) are vulnerable to attack by *F. auricularia* during emergence and development. They also suggest a phenotypic capacity of this species to adapt different phenology after introduction into a novel environment. © 2020 The Authors. *Pest Management Science* published by John Wiley & Sons Ltd on behalf of Society of Chemical Industry.

## Graphical abstract

The lifecycle of *Forficula auricularia* in grain crops is presented. We found that the Australian populations of *Forficula auricularia* may develop much earlier than observed in comparable North American studies.

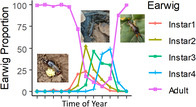

## 1 INTRODUCTION

Globally distributed invertebrates offer unique opportunities to study how species adapt to different environments and habitats. To date, the majority of studies have focussed on global species of economic and agricultural importance^1^ and factors that impact their ability to successfully invade new regions (e.g. pathways^2^ and physiological traits^3^). However, understanding how species change their lifecycles, diet, species interactions and important behaviours once established in a new region is also important. The European earwig, *Forficula auricularia* L. (Dermaptera: Forficulidae), native to Europe, western Asia and Northern Africa,^4^ is now found in most temperate regions globally. *Forficula auricularia* was introduced to Australia over 170 years ago,^5^ yet little is known about how this species survives across different geographies (but see Tourneur & Meunier^6^). *Forficula auricularia* is widely distributed across southern Australia, has strong spatial overlap with major grain‐growing regions and is principally restricted by aridity.^7^ *Forficula auricularia* is considered a generalist, feeding on soft plant tissue, fungi, organic matter and other invertebrates.^4^ It is an important pest in Australian grain crops where earwigs feed on emerging crop seedlings, resulting in reduced and irregular crop densities.^8^, ^9^, ^10^ Crop feeding damage is being reported at an increasing rate in canola, wheat, barley, oats, and lupins, often during late autumn and winter when large numbers of earwigs are observed in fields.^11^

Within Australia, farmers attempt to control *F. auricularia* within grain crops by applying insecticide seed treatments, chemical baits, foliar insecticides, or by removing crop habitats (i.e. crop stubble) that favour the survival of this species.^12^, ^13^, ^14^ The pest status of *F. auricularia* is not necessarily straightforward. In many cases, large numbers of *F. auricularia* are observed in crop fields but cause little or no discernible feeding damage to growing plants.^11^ Furthermore, similar to other parts of the world, Australian populations of *F. auricularia* can be predatory, feeding on aphids, caterpillars, mites and other soft‐bodied arthropods.^15^, ^16^, ^17^ In fact, *F. auricularia* is a well‐recognized beneficial predator in apple, pear and kiwifruit orchards,^5^, ^18^, ^19^, ^20^ vineyards,^21^ and has even been considered beneficial in some grain crops.^22^, ^23^, ^24^, ^25^

To manage invertebrates of agricultural significance it is important to understand their lifecycle. The susceptibility of a species to certain interventions can change substantially at different points in their lifecycle. For example, species such as *F. auricularia*, which are cryptic and spend a considerable amount of time underground,^26^ are unlikely to be affected by foliar insecticide applications until they emerge and come into contact with that insecticide. Understanding the lifecycle of *F. auricularia* within orchards has enabled farmers to predict when earwigs are likely to suppress pest invertebrates. Using this knowledge, farmers are able to make informed management decisions (i.e. spraying insecticides, tilling) that reduce the impact on earwig numbers, thus maximizing their beneficial activity.^5^, ^18^, ^27^

*Forficula auricularia* is hemimetabolous, undergoing incomplete metamorphosis with three life stages: the egg, nymphal stage and adult.^4^ It is a sub‐social insect, whereby females tend their clutch of eggs and provide food and protection to early instars.^26^ After hatching, *F. auricularia* nymphs undergo a series of moults until adulthood is reached. There are four nymphal stages of development, called instars.^4^ The lifecycle of *F. auricularia* has been examined in cool and temperate regions in the Northern hemisphere,^28^, ^29^, ^30^, ^31^, ^32^, ^33^, ^34^, ^35^, ^36^, ^37^, ^38^, ^39^, ^40^ with information recently garnered from Australian orchard environments^41^, ^42^ and New Zealand.^20^, ^43^ Oviposition occurs within subterranean nests excavated by the female, in the northern hemisphere, this typically occurs from late summer to spring depending on the region. The female cares for her offspring until they reach second instars, before summer.^26^ By the third instar, earwigs tend to move onto trees, where they moult into fourth instars by mid‐summer. Two reproductively isolated lineages of *F. auricularia* have been identified in Europe and North America; clade A which normally produces one brood per year (and occasionally two^44^) and clade B which typically produces two broods per year^45^ (but occasionally three^46^ or one broods have been observed^45^) It has recently been confirmed that Australia harbors clade B, with no evidence of clade A occurring.^5^

Our objectives for this study are to aid industry in understanding likely risks posed by *F. auricularia*. Specifically, we aim to better understand the production of multiple broods within a season, and the timing of each life stage. We undertook extensive field collections of *F. auricularia* at multiple locations across southern Australia to elucidate the lifecycle of *F. auricularia*. Degree‐day models were developed to further characterize the observed temporal patterns. This data set allowed us to compare the consistency of the lifecycle across different locations. Being established for approximately 170 years in Australia, the determination of lifecycles facilitated the testing of a second question. Does *F. auricularia* have a phenotypic capacity to adapt its phenology after introduction into a new environment? Results from different trapping methods are presented and discussed.

## 2 MATERIALS AND METHODS

### 2.1 Site details

**Figure 1:**
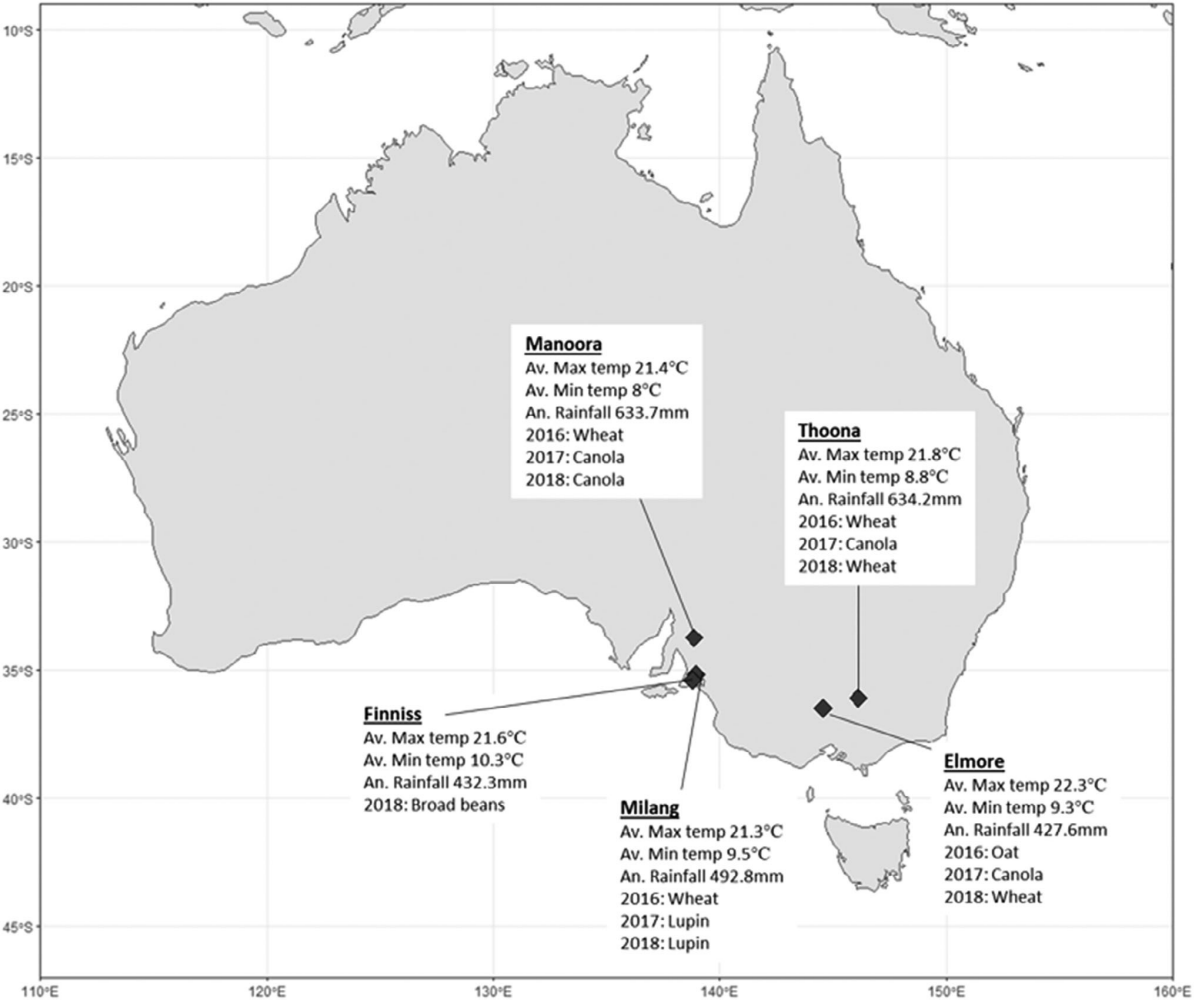
Map of Australia showing the location, climatic data and crop type at each field site used in this study.

Five fields were selected across south‐eastern Australia where grain crops are grown annually. These fields were separated by at least 50 km and were chosen because: (i) growers had reported the presence of earwigs within these fields in previous years, and (ii) they represented different agro‐ecological regions in terms of climate and geography (Fig. 1). Two fields were located in the state of Victoria (VIC), and three in South Australia (SA). All farms use minimum tillage practices, with crop seeds directly sown into fields containing stubble from the previous year's crop. No insecticide sprays were applied within 50 m of the earwig monitoring locations at any field site for the duration of this study.

### 2.2 Field sampling

**Figure 2:**
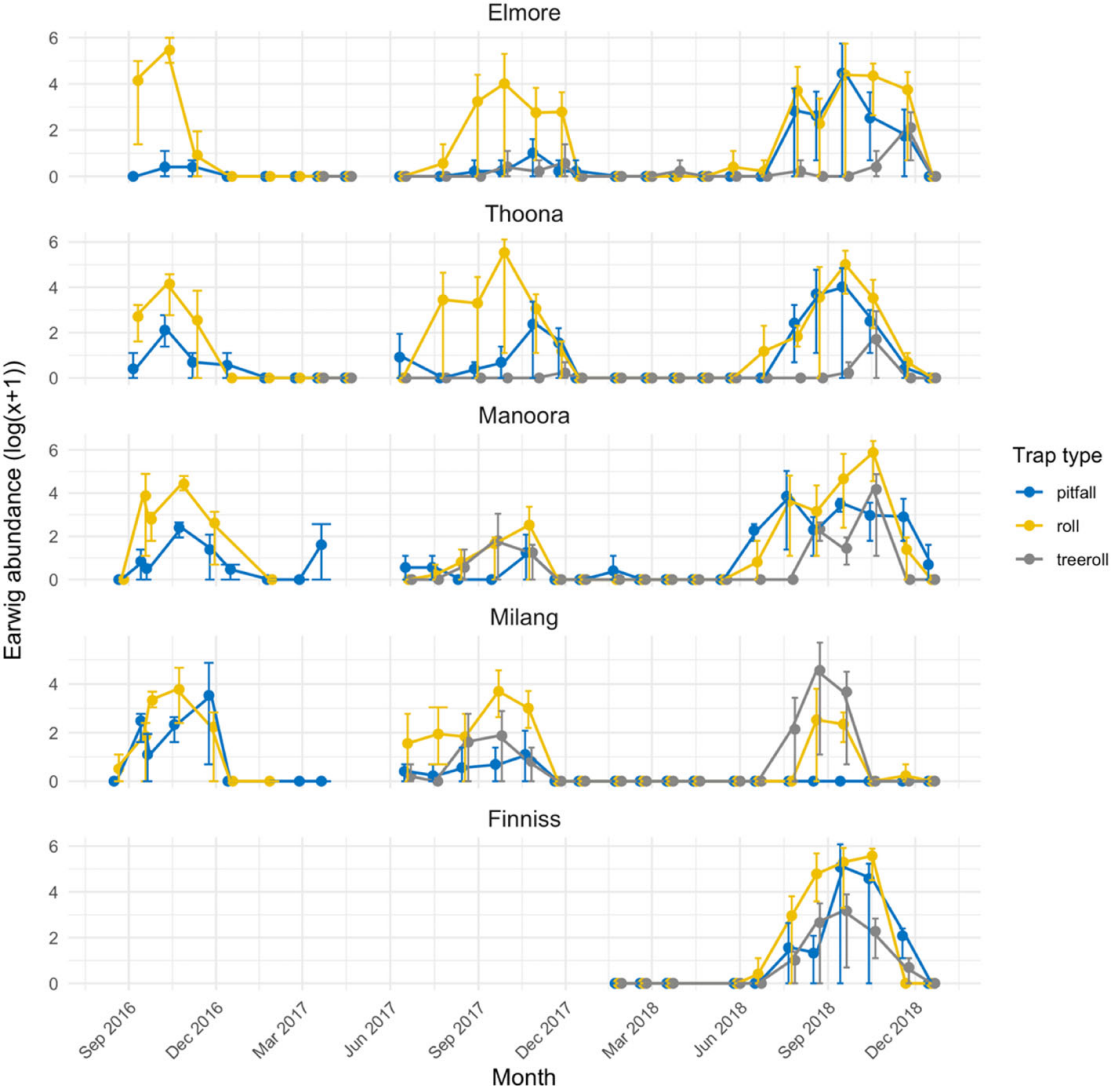
Log mean abundance of *Forficula auricularia* juveniles (all instars combined) collected from the three trap types at each field site. Total individuals for each site are: Elmore = 3190, Thoona = 3162, Manoora = 3582, Milang = 1459, Finniss = 3661. Error bars show the range of the data. Note different scales used for each site.

Earwig sampling was undertaken at each of the field sites at monthly intervals as described below, although sampling was missed in some months (see Table 1 and Fig. 2 for full details). Every month, we deployed two types of traps in the field: cardboard rolls and pitfall traps. These trapping techniques were used to reduce the risk of capturing data reflecting trap inefficiencies at certain times of the year rather than reflecting real changes in *F. auricularia* abundances. Pitfall traps were chosen because they are effective at capturing earwigs during times of soil surface activity (e.g. foraging for food at night).^17^ Cardboard roll traps were selected because they are effective at capturing earwigs during times of relative inactivity (e.g. sheltering during the day).^33^

**Table 1:** Total number of all earwig species collected by trap type at each field site

	Total number of earwigs			
Field site (state)	Pitfalls	Rolls‐ground	Rolls – trees*	Total
Elmore (VIC)	1213 (1189)	5117 (5113)	5371 (5361)	11 701 (11663)
Thoona (VIC)	996 (866)	2905 (2872)	1156 (1155)	5057 (4893)
Finniss (SA)	1328 (1306)	2759 (2750)	278 (275)	4365 (4331)
Manoora (SA)	1344 (1174)	3026 (2981)	414 (388)	4769 (4543)
Milang (SA)	573 (456)	770 (728)	624 (616)	1967 (1800)
Average number of earwigs per trap	84.3	287.7	130.7	

The number in parenthesis is the number of *F. auricularia* trapped. Elmore and Thoona were sampled from Sept‐2016 to Dec‐2018 (27 observations each), Manoora and Milang were sampled from Aug‐2016 to Dec‐2018 (27 observations each), and Finniss was sampled from Jan‐2018 to Dec‐2018 (12 observations).

^*^ Only eight rolls were used in the trees, whereas 12 ground rolls and 12 pitfall traps were used at each site. Tree rolls were only used from March 2017, whereas pitfalls and ground rolls were used from the beginning of each collection period.

Pitfall traps consisted of a polyvinyl chloride (PVC) pipe placed in the ground, flush with the ground surface. Vials 45 mm in diameter and 120 mL volume, containing 60 mL of 100% propylene glycol, were placed inside the PVC pipe and left open for 7 days. At each field site, pitfall traps in groups of three (2 m apart) were installed at four locations, three within the paddock (> 30 m from the edge), and one on the edge of the field (i.e. total 12 pitfall traps per site). Trap locations were set >30 m apart. After 7 days in the field, pitfall traps were collected, transported back to the laboratory and stored at 4 °C until processing.

Cardboard rolls consisted of single sided corrugated cardboard, 250 mm in width, rolled to form 50 mm diameter cylinders with longitudinal corrugations. The rolls were inserted into a 200 mm length of 50 mm diameter PVC pipe. Three rolls were placed within an inter‐row (and parallel to the crop/stubble row), approximately 2 m from the pitfall traps (i.e. 12 ground rolls per field). As with the pitfall traps, ground rolls were left in the field for 7 days before collection.

From March 2017 onwards, we additionally placed two cardboard rolls within the canopy of four trees within each field site (i.e. eight tree rolls per site) following the approach described by Moerkens *et al*.^30^ We did this to test if high earwig numbers in this environment correlated with high numbers in the field, as might be expected if there was migration from trees to the field and *vice versa*. The trees were either native (*Eucalyptus* spp. and *Casuarina* spp.) or exotic species (*Schinus molle* and *Populus* spp.) or a combination of both, depending on what vegetation immediately surrounded each field. Additionally, in Victoria on each sampling day, the presence of earwig nests around the trapping location at each field site was observed by looking under rocks, soil clods and crop stubble. This was undertaken by visually searching for approximately 10 min in a 10 m radius around each trap location. The presence of nests with eggs were recorded.

Micro‐climatic data was recorded at each site using Hobo 4 channel micro‐data loggers (H21). Data loggers were set to record data at 30 min intervals and were deployed in each field. These were equipped with a sensor for relative humidity and temperature (S‐THB‐M002) and were placed at ground level. To provide temperature information for any missing observations, weather station data (maximum and minimum temperatures, rainfall) at each location was accessed from the nearest Bureau of Meteorology weather station using the ‘bomrang’ package^47^, ^48^ in R 3.6.1.^49^ The distance between each site and the nearest weather station was: Thoona 22.1 km, Elmore 37 km, Manoora 27.2 km, Milang 15.1 km and Finniss 12.5 km.

### 2.3 Identification and determination of earwig life stages

Within 3 days of collection, pitfall samples were rinsed in tap water, all earwigs removed using fine tweezers and placed into Eppendorf tubes with 100% ethanol. Earwigs collected in the cardboard rolls and tree rolls were anesthetized with CO_2_ gas and then removed by unrolling the cardboard sheets and transferring individuals into Eppendorf tubes with 100% ethanol. All *F. auricularia* individuals were separated from the other Dermapteran species that were captured. This was achieved using morphological characteristics under ×40 magnification as described in Crumb,^4^ with species determinations verified by molecular methods as needed (results presented in Stuart *et al*.^50^). Individual adult and nymphal stages were categorized using head width, the number of antennal segments and wing bud development^4^ (Supporting information, Figs. S1 and S2). Nymphal stage data was pooled at Milang, Manoora and Finniss at each collection time, hence we cannot present variation in mean abundances at this level, only when separated as adults and juveniles. As earwigs are sexually dimorphic, sex was identified using the shape of the cerci and categorized into male, female and gynandromorphs.

We checked for the presence of eggs within a subset of up to 10 adult female *F. auricularia* from each replicate trap on all sampling dates between March 2018 and December 2018. This was undertaken by excising the ventral side of the female abdomens. This occurred for earwig samples collected at all five field sites.

### 2.4 Statistical analysis and degree‐day modelling

To compare *F. auricularia* caught in different traps, we first determined the number of juvenile and adult earwigs separately for each trap type at each site and pooled across traps for each collection period between when traps were placed out and then retrieved (we refer to the time of retrieval as the sampling date) (Fig. 2). We then computed non‐parametric Spearman rank correlations between sampling dates to determine if traps collected similar life stages and numbers of earwigs. Adults were usually retrieved by at least one of the trap types at each collection period. However, there were many periods when juvenile earwigs were not captured (see Fig. 2 for these periods, which were mainly around January to June). To compare trap types, we therefore computed the correlations by both including and excluding periods when no juvenile earwigs were captured. To determine if one trap type consistently collected more earwigs than another, we used Friedman tests to compare trap types across collection periods. Where a significant or marginally significant difference was detected in the Friedman test, we undertook Wilcoxon tests to compare trap types paired within each collection period (i.e. one data point for each trap type within a collection period). These analyses were performed using IBM SPSS Statistics (version 26).

Degree‐day modelling was performed to better understand the phenology of *F. auricularia* under Australian conditions. Moerkens *et al*.^27^ determined developmental rates for each stage: hatching, moult 1, moult 2, moult 3 and moult 4. From those developmental relationships, we were able to obtain the upper and lower development rate parameters for each life stage and the number of degree‐days required to complete the development of each stage. These parameters were then used to estimate when later instars would be present in the field, given the observation of first instars. To do this, we used the temperature observations taken half‐hourly to determine the daily *T*_*min*_ (minimum temperature), *T*_*max*_ (maximum temperature) and *T*_*a*_ (average temperature) values at each field site. Missing values were patched in from the weather station data to provide complete temperature vectors. We then calculated the degree days accumulated for each day, using the corresponding parameters (upper and lower thresholds) estimated in Moerkens *et al*.^27^ for each stage using a simple sine method.^51^ The duration of each life stage was estimated using simulations of 1000 individuals from the date of a first instar observation. We randomized the start day of the simulation across 5 days prior and including the respective first instar observations. For each life stage, we included stochasticity by taking the error associated with the estimated parameters and allowing the values per simulation to be drawn from within this range. As it is not possible to link each observation of an instar to a particular cohort in the field, we present the results with all data overlaid on each model. This provides a conservative estimate of the expected duration of instars at each field site.

Simulations were performed using functions written in R (see Supporting information). All figures were produced using ggplot2.^52^

## 3 RESULTS

### 3.1 Earwig abundance and trapping methods

In total, 27 859 earwigs of multiple species were captured, of which 27 230 were *F. auricularia*, 193 were *Labidura truncata*, 82 were *Nala lividipes*, and the remaining were unknown species of *Anisolabis* and *Gonolabis*. Victoria accounted for 60% of all samples, with 98% identified as *F. auricularia* while 96% of individuals from South Australia were *F. auricularia* (Table 1). This supports previous studies that have found *F. auricularia* to be the most abundant earwig species in grain crops in southern Australia.^10^, ^50^ We found head capsule width to be the most consistent trait in determining life stage, as occasionally the antennae of earwigs were damaged and missing segments. Across all sites, the average head capsule width measured across more than 2500 individuals was 0.92 (± 0.01) mm for first instars (n = 215), 1.29 (± 0.02) mm for second instars (n = 505), 1.53 (± 0.01) mm for third instars (n = 892), and 1.84 (± 0.01) mm for fourth instars (n = 908) (Supporting information Fig. S1), which is highly congruent with previous studies in the northern hemisphere.^4^

All trapping methods were successful in capturing large numbers of *F. auricularia* although the average number of earwigs collected per trap was more than twice as high for the ground rolls than the other trap types (Table 1). Most correlations (14 out of 15 cases) across trap type were positive for adults, and four correlations were significant (Table 2). A comparison of numbers caught in traps across the collections at each field site indicated no significant differences between trap type in the number of adults collected (Table 3). For juveniles, we failed to collect earwigs on many occasions (Fig. 2) and therefore analyzed the data in two ways, by either including or excluding collection periods where no juveniles were caught. When they were included, correlations between numbers caught per trap were positive in 14 out of 15 cases, including 12 significant correlations (Table 2). When they were excluded, correlations were also positive in 14 out of 15 cases, and four correlations were significant (Table 2), so the inclusion or exclusion of juveniles did not affect the extent to which trap type provided similar patterns of earwig populations. The total number of juveniles caught per trap type differed significantly at three sites, while at Milang there was a marginally non‐significant difference (Table 3). At sites where differences were detected among trap types, pairwise comparisons indicated that ground rolls captured more juveniles than other trap types (Table 3).

**Table 2:** Non‐parametric correlations (Spearman's rho) between trap type and the number of *F. auricularia* collected at each field site

Field site	Life stage (trap number^†^)	Pitfalls – ground rolls	Pitfalls – tree rolls	Ground rolls ‐ tree rolls
Elmore	Adults (27/21)	0.348	0.675**	0.565*
	Juveniles ‐ all (27/21)	0.748**	0.493*	0.577*
	Juveniles – zero excl. (17/14)	0.533*	0.216	0.447
Thoona	Adults (27/22)	0.431*	0.277	−0.014
	Juveniles ‐ all (27/22)	0.726**	0.547**	0.398
	Juveniles – zero excl. (16/13)	0.397	0.540	0.235
Finniss	Adults (11/11)	0.416	0.516	0.064
	Juveniles ‐ all (11/11)	0.659*	0.951**	0.805**
	Juveniles – zero excl. (5/5)	0.300	0.600	0.900*
Manoora	Adults (28/20)	0.309	0.040	0.596**
	Juveniles ‐ all (28/20)	0.737**	0.462*	0.710**
	Juveniles – zero excl. (18/13)	0.631*	0.357	0.232
Milang	Adults (27/20)	0.359	0.214	0.075
	Juveniles – all (27/20	0.787**	0.427	0.000
	Juveniles – zero excl. (13/9)	0.610*	−0.293	0.310

For adults, all collection periods are included. For juveniles, both the correlations for all traps (all) and those only including months where juvenile earwigs were captured in at least one trap (zero excl.) are given. The first number is the number of comparisons made for the pitfall – ground roll comparison, the second is the number for both comparisons involving tree rolls.

^*^ *P* < 0.05.

^**^ *P* < 0.01.

^†^ Sample sizes differ between sites because of differences in the length of time that traps were out and when juveniles were absent from the different field sites.

**Table 3:** Comparisons between trap types based on the total numbers of juvenile or adult *F. auricularia* collected in each period (excluding months when no earwigs were collected for any of the trap types)

Field site	Juveniles						Adults	
	N	Mean ranks			χ^2^ (Friedman test, df = 2)	Significant pairwise differences between trap types (z from paired Wilcoxon test)	N	χ^2^ (Friedman test, df = 2)
		p	gr	tr				
Elmore	14	1.86	2.64	1.50	10.510***	gr > p (2.767), gr > tr (3.170)	21	0.625
Thoona	13	2.23	2.65	1.12	17.429****	gr > p (2.379), gr > tr (3.059), p > tr (2.940)	22	0.963
Finniss	5	1.9	2.6	1.5	3.263		10	2.923
Manoora	13	2.19	2.35	1.46	6.292**	gr > tr (2.805)	20	4.324
Milang	9	1.44	2.5	2.44	5.353*	(gr > p) (2.197)	13	0.311

All three trap types were compared by Friedman tests, followed by paired Wilcoxon tests in cases when Friedman tests indicated differences between trap type. gr, ground roll, p, pitfall, tr, tree roll. Sample sizes for comparisons (N) are provided for both juveniles and adults, while mean ranks from Friedman tests are also provided for juveniles.

^*^ *P* = 0.07.

^**^ *P* < 0.05.

^***^ *P* < 0.01.

^****^ *P* < 0.001.

**Figure 3:**
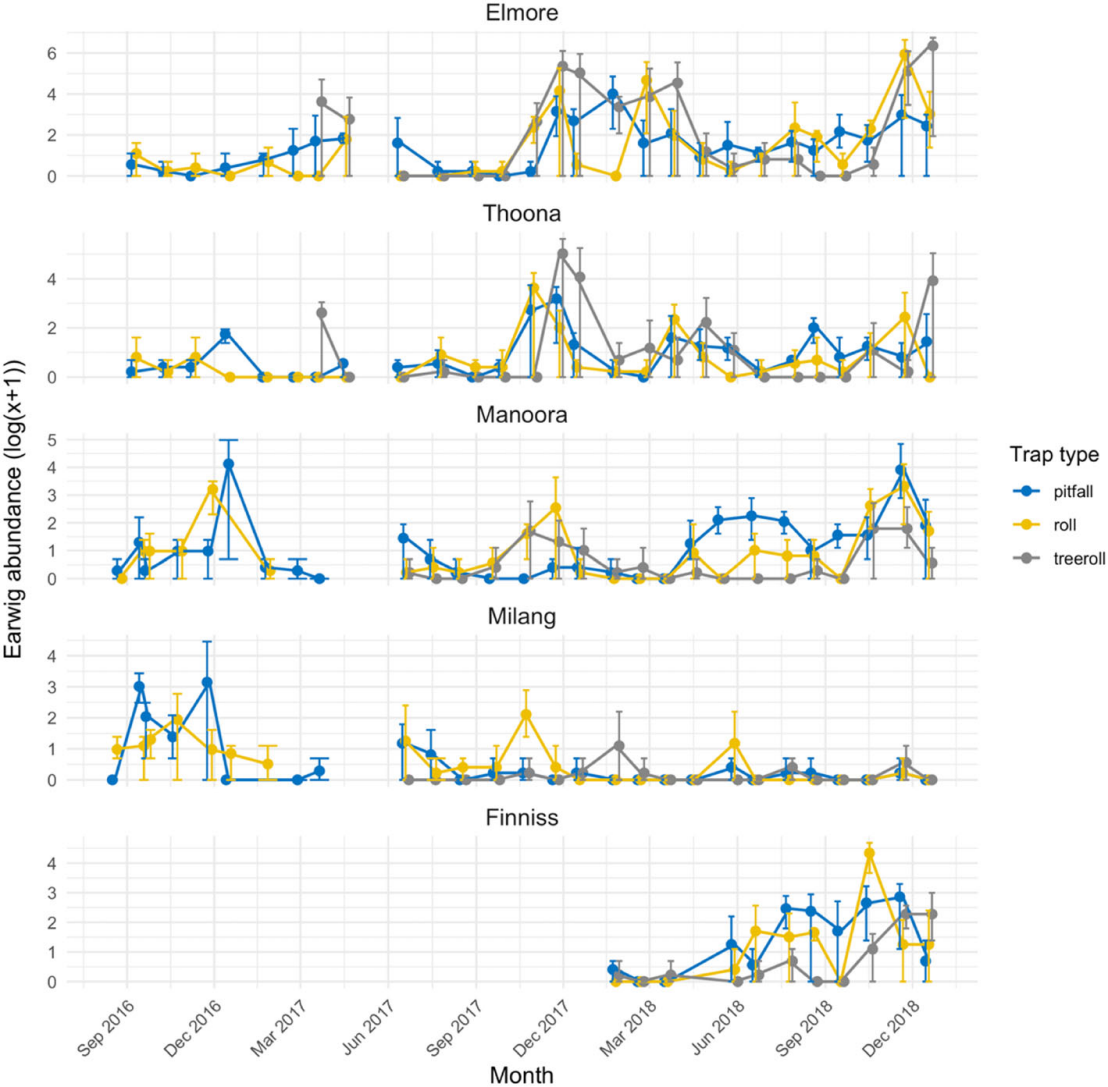
Log mean abundance of *Forficula auricularia* adults collected from the three trap types at each field site. Total individuals for each site are: Elmore = 8473, Thoona = 1731, Manoora = 1150, Milang = 319, Finniss = 696. Error bars show the range of the data. Note different scales used for each site.

When examining the data across time, there were further differences in trapping success between pitfalls, ground rolls and tree rolls, with differences most noticeable when only considering juvenile earwigs (Figs. 2 and 3). Pitfall traps collected most *F. auricularia* from mid‐autumn to mid‐winter, while ground rolls were more successful from mid‐winter to late spring. The tree rolls collected more *F. auricularia* during spring and early summer (Fig. 2).

The synchrony of the peak abundance of juvenile *F. auricularia* captured in our traps was relatively consistent across all sites and years (Supporting information Fig. S3). *Forficula auricularia* instars were absent or in very low abundances from late summer to early winter. The timing of peak adult captures was variable between sites, and in some cases, within sites across years (Fig. 3).

**Figure 4:**
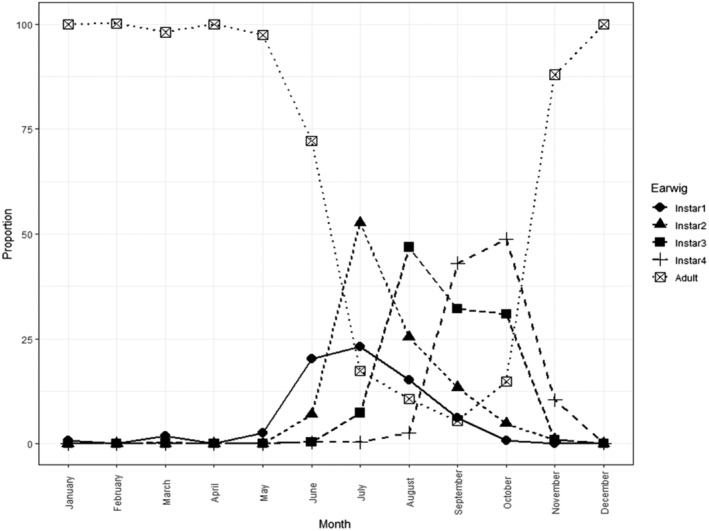
Generalized phenology of *Forficula auricularia* in south‐eastern Australia (all years, sites and trapping methods combined). The proportions shown are the mean proportions across field sites and years for each month.

By combining collection data from all fields, trapping methods and years, an annual phenology for *F. auricularia* can be depicted for south‐eastern mainland Australia (Fig. 4). Peak proportions of successive life stages are: first instar (June/July), second instar (July), third instar (August/September) and fourth instar (September/October). Adult proportions remain high from November until June (Fig. 4). Eggs were present in dissected females from March onwards at Elmore and Thoona, and April onwards at Finniss and Manoora (Table 4). No eggs were recorded from dissections of females collected at Milang, although the total number of samples examined was very low at that site (n = 2). Very few female *F. auricularia* with eggs were found after August, irrespective of site (Table 4). We had insufficient data to formally test for differences between trap type, but we have an indication that proportions of females with eggs may be fairly consistent between traps (Supporting information Fig. S4). Earwig nests were found at both Victorian sites, typically located under rocks and clods of dirt. These were predominantly observed from June to July and typically contained 60–80 eggs per nest.

**Table 4:** Percentage of female *F. auricularia* that contained eggs upon dissection at each field site in 2018

Field site						Month						
	Jan	Feb	Mar	Apr	May	Jun	Jul	Aug	Sep	Oct	Nov	Dec
Elmore	—	—	74.5 (51)	100 (11)	100 (8)	72.7 (11)	44 (25)	29.2 (24)	18.2 (11)	0 (45)	0 (94)	0 (75)
Thoona	—	—	79.4 (34)	92.3 (26)	70 (10)	0 (1)	40 (5)	18.2 (22)	0 (2)	7.7 (13)	0 (20)	0 (29)
Finniss	—	—	—	90.3 (31)	75 (4)	66.7 (15)	34.8 (23)	18.2 (22)	0 (3)	—	—	—
Manoora	—	—	—	66.7 (6)	0 (4)	33.3 (15)	33.3 (18)	71.4 (7)	0 (5)	0 (3)	100 (1)	—
Milang	—	—	—	—	—	—	—	0 (2)	—	—	—	—

Up to 10 females were dissected per trap type and sample date. The number in parenthesis is the total number of females dissected at each site for each month. A dash (−) indicates no female dissections occurred from samples collected during the month. Data from all trap types are combined.

### 3.2 Degree day model

**Figure 5:**
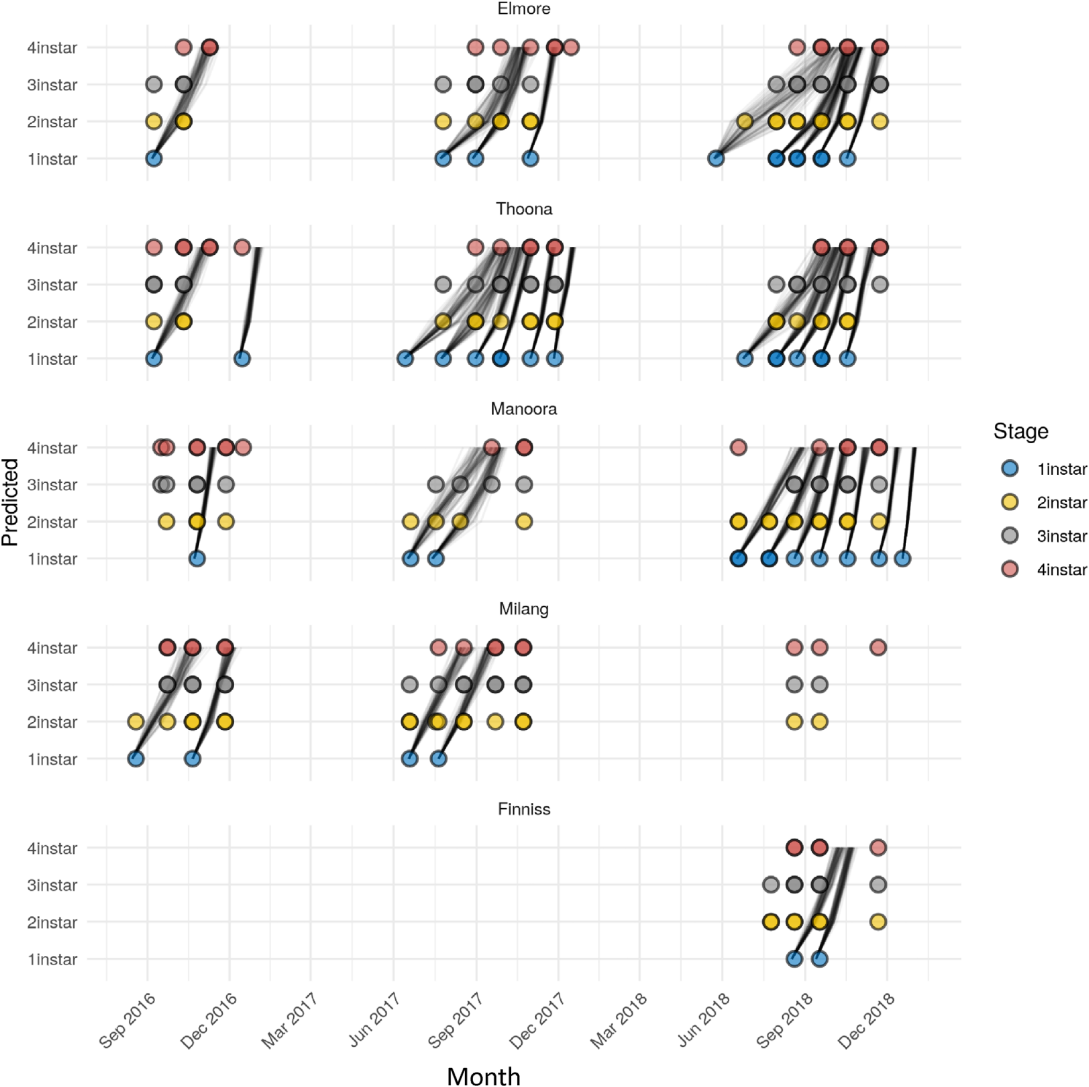
Day degree models for *Forficula auricularia* run at each sampling date that held an observation of a first instar. Circles depict the presence of each juvenile life stage collected at each sampling point. Lines represent a single model output and depict the first date at which each life stage was predicted in the models.

The degree‐day models show the relative development rates of *F. auricularia* at each field site and likely occurrence of each life stage at different times across the sampling period (Fig. 5). These outputs are based on the presence of life stages at each site, so model predictions outside of our observation range could be due to earwigs being present in the field but not being collected at that time point. Overall, the development of *F. auricularia* takes longer for first instars (observed in June) compared with later instars, although this varies somewhat between locations. The South Australian sites, Manoora and Milang, appear to have faster development times over the winter period (June–August) compared with the Victorian sites, Elmore and Thoona. As the seasons become warmer (September and October), development rates are predicted to be quicker, with development in the later months (November and December) becoming rapid (Fig. 5). The increase in degree days after winter allows for full development of juvenile earwigs by mid‐summer, even if the first instars only hatch at the end of spring.

### 3.3 Comparison of lifecycle in Australia with northern hemisphere

We compared the seasonal timing of *F. auricularia* life stages found in this study with patterns recorded in the northern hemisphere, specifically those from North America and Europe.^26^, ^27^, ^29^, ^35^, ^36^, ^37^, ^38^, ^39^, ^40^ After adjusting for the 6 month seasonal differences between the northern and southern hemispheres, we find the lifecycle of *F. auricularia* is completed much earlier in our Australian regions than the northern hemisphere, despite similar dates for egg‐lay (Table 5). First instars hatching from eggs appeared early in winter, almost 6 months earlier than in the northern hemisphere. This difference was shorter with later life stages, but we saw the adult moult happen in early summer which was still 3 months earlier than what was reported in the northern hemisphere.

**Table 5:** The seasonal timing of key life stages of *F. auricularia* recorded in this study and from published studies in the northern hemisphere (Europe or North America)

Life stage	Our study	Northern hemisphere (and key references)
First clutch egg lay	Late Autumn	Summer (Tourneur and Meunier 2019, Dib *et al*. 2017) Winter (Lamb 1976)
Second clutch egg lay	Unknown	Spring (Tourneur and Meunier 2019, Dib *et al*. 2017)
First instar	Early winter	Spring (Dib *et al*. 2017) Late spring (Lamb 1976)
Second instar	Winter	Winter, spring and summer (Lordan *et al*. 2015) Late spring (Dib *et al*. 2017, Lamb 1976)
Third instar	Spring	Winter, spring (Lordan *et al*. 2015) Spring (Dib *et al*. 2017) Early summer (Lamb 1976, Gobin *et al*. 2008)
Fourth instar	Spring	Spring (Lordan *et al*. 2015) Spring, summer (Dib *et al*. 2017) Mid‐summer (Moerkens *et al*. 2011, Gobin *et al*. 2008)
Adults	Summer	Late spring (Romeu‐Dalmau *et al*. 2012, Lordan *et al*. 2015) Summer (Lamb and Wellington 1975, Lordan *et al*. 2015, Romeu‐Dalmau *et al*. 2012, Saladini *et al*. 2016, Dib *et al*. 2017) Late summer (Gobin *et al*. 2008) Autumn (Lamb and Wellington 1975, Romeu‐Dalmau *et al*. 2012)

## 4 DISCUSSION

Given *F. auricularia* was introduced over 170 years ago^5^ and is now widely distributed across southern Australia,^7^ there is relatively little known about the factors that influence the timing of key events in its lifecycle. By using different trap types, in different locations and in multiple fields, we are able to make inferences about the lifecycle and ecology of *F. auricularia* in Australia, and thereby better understand the risk posed by this species to grain crops, and perhaps understand the potential beneficial value they might also have in these systems.

Using multiple trap‐types, our data show the lifecycle of *F. auricularia* in Australian arable ecosystems is characterized by the overlapping development of immature life stages, with first/s instars observed throughout winter and spring (June–October), third/fourth instars peaking later in spring (August–November), and adults found year‐round, but most prevalent throughout summer and autumn. As expected, there was some variability in temporal patterns between sites. Quarrel *et al*.^41^ presents weekly data showing, on many occasions, a distinct second (albeit smaller) peak in abundances for immature stages of *F. auricularia* within apple orchards. This occurred approximately 1 month after their numbers initially peak. Interestingly, we did not observe distinct peaks for immature stages; instead, there was overlap in life stages across a number of months. This indicates variable timing of the second brood at our sites. Three nests at Thoona and one nest at Elmore (each containing less than 30 eggs) were observed in late October 2018. This occurred shortly after a large rainfall event that followed a prolonged dry period and is highly likely to represent a second brood at these sites. However, these detections were not followed by observations of corresponding juveniles in the field. This may reflect the temporal nature of our sampling, which occurred monthly. As demonstrated by our degree‐day modelling, *F. auricularia* is able to rapidly develop through multiple life stages under warmer conditions, which occurs from late spring to early autumn in southern Australia. It is possible, weekly sampling intervals during this period would have detected instars from those eggs observed in October.

As with the initial invasion into North America, *F. auricularia* has likely adapted to conditions in Australia that are not present in its native range.^5^, ^7^ There are likely to be considerable advantages to non‐synchronized brood production in temperate agricultural environments, given earwigs experience considerable changes in habitat quality from year to year. The nutritional resources available to the juvenile earwigs will differ with crop type, plant growth stage, the presence of other invertebrates (earwig prey such as aphids) and climate.^15^, ^53^ *Forficula auricularia* produce different chemical signals depending on their nutritional state.^54^ Mas and Kolliker^54^ observed that females groomed their offspring significantly more when exposed to the well‐nourished chemical signal, but were aggressive to the brood when exposed to a poorly nourished signal. This is likely to affect the time females spends with her first brood, and subsequently the timing of the second brood.

Our study indicates an earlier developmental cycle of Australian *F. auricularia*, by approximately 3 months (when adjusted for seasons), when compared with climates with much cooler winters, such as in northern America and Europe. This is likely due to the ability of earwigs to accumulate degree days much faster in Australian winters which are relatively mild. The lower development threshold for *F. auricularia* eggs is 5.3 °C,^27^ which is lower than the lowest soil temperature recorded at our field sites. Lower soil temperatures in those regions with cold winters prohibits the development of *F. auricularia* eggs,^27^ whereas at our field sites, eggs are able to develop consistently following oviposition. Perhaps this is why our findings are similar to those observed in Southern Europe,^37^, ^38^, ^39^ where winter temperatures are most analogous to Australia.

Our findings also demonstrate significant differences in relative abundances of *F. auricularia* based on trapping method, but also a positive correlation between trap type and numbers collected. This is not surprising given the nature of each trap; pitfall traps kill earwigs that walk close enough to fall in, whereas cardboard rolls provide an attractive refuge that encourage congregation of earwigs but allows them to move freely in and out. The high catches in pitfall and cardboard roll traps (on the ground) relative to cardboard rolls in trees in late autumn‐winter (May to July) may reflect the higher ground activity of earwigs as they move around crop fields and start excavating nests.^26^ The relatively high catches in tree rolls at the fourth instar and adult stages during late spring–summer is consistent with previous studies,^41^ and aids our understanding of where *F. auricularia* move post‐grain harvest. Based on our findings, farmers monitoring for earwigs in grain crops should ideally use a combination of ground rolls, pitfall traps and tree rolls throughout the year. However, if resources are limited, ground traps (either pitfalls or cardboard rolls) should be used from autumn to late spring, and tree rolls used from spring to autumn.

Previous authors have recommended using a combination of trap types, including cardboard rolls, as we have done in this study.^19^ It is important to note however, the numbers of *F. auricularia* reported here are a function of individual activity recorded by the different trap types and the population density in each field. It is unclear how earwig behavior influences the likelihood of capturing individuals in each trap type. For example, the aggregation behaviour of *F. auricularia* appears to be strongly influenced by the cuticular hydrocarbons produced by individual earwigs.^55^, ^56^ Thus, at certain stages of the lifecycle, cardboard rolls already containing earwigs are likely to attract more earwigs than empty rolls.^57^ Additionally, earwigs may be less likely to use the cardboard rolls if there is alternative shelter in the surrounding area.^43^ Extrapolation of trap catches to actual numbers of *F. auricularia* in a given field must therefore by made with caution. The development of a crop growth model (with alternate planting date options) linked to a *F. auricularia* model may be a more useful tool for farmers seeking to understand the risk of earwig damage prior to sowing. However, given crop damage from earwigs is challenging to document due to their varied feeding habits,^4^, ^16^, ^17^, ^58^ the development of an action threshold linked to earwig abundance and crop growth model may be unachievable, at least without further manipulative studies.

In summary, this study provides further evidence that *F. auricularia* is well adapted to the Australian agricultural environment (see also Hill *et al*.^7^). The timing of the majority of egg hatching aligns closely with the emergence of winter crops across a large part of southern Australia. This includes canola, lucerne and pulse crops, which are known to be vulnerable to attack from *F. auricularia*, especially as young seedlings.^58^ Our results indicate earwigs can persist within agricultural fields for the majority of the winter‐cropping season in Australia, with juveniles developing into adults by harvest time in late spring. Furthermore, we found evidence that a second brood can be produced very late in the season, just before harvest in Australia's grain growing regions. It remains unclear how much this second brood contributes to the overall population dynamics. Further research is warranted to understand the conditions (abiotic and biotic) influencing the production of a second brood in Australian *F. auricularia* and the degree of plasticity around this trait.

## SUPPORTING INFORMATION

Data and R code to run simulations and generate Figs. 2, 3 and 5 is available at figshare: 10.6084/m9.figshare.12580757.

## Supporting information


**Appendix** **S1.** Supporting Information.
