# Supplementary material for: Lifecycle of the invasive omnivore, Forficula auricularia, in Australian grain growing environments
Source: Pest Manag Sci. 2020 Dec 22;77(4):1818–28. doi: 10.1002/ps.6206 (PMC7986395; doi:10.1002/ps.6206)
Supplement: Supplementary file 1 — Appendix S1. Supporting Information. [file PS-77-1818-s001.docx]

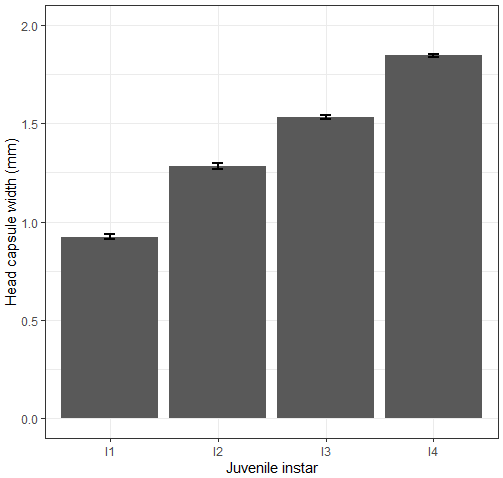


**Suppl. Fig. 1** Earwig head capsule width against each juvenile life stage for *Forficula auricularia* (all field sites combined).


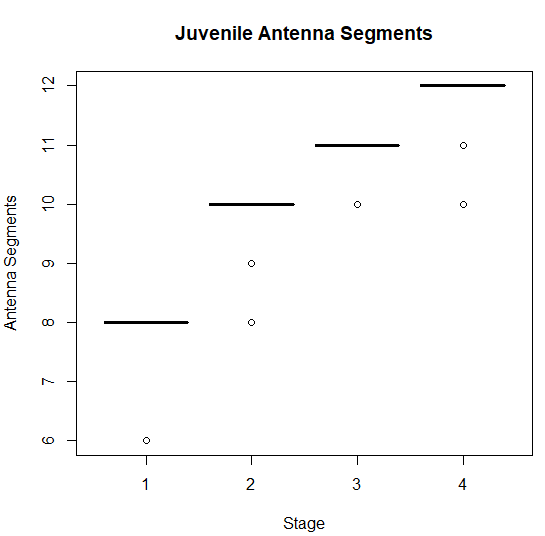


**Suppl. Fig. 2** Boxplots showing the number of antennal segments against each instar life stage for *Forficula auricularia* (all field sites combined).


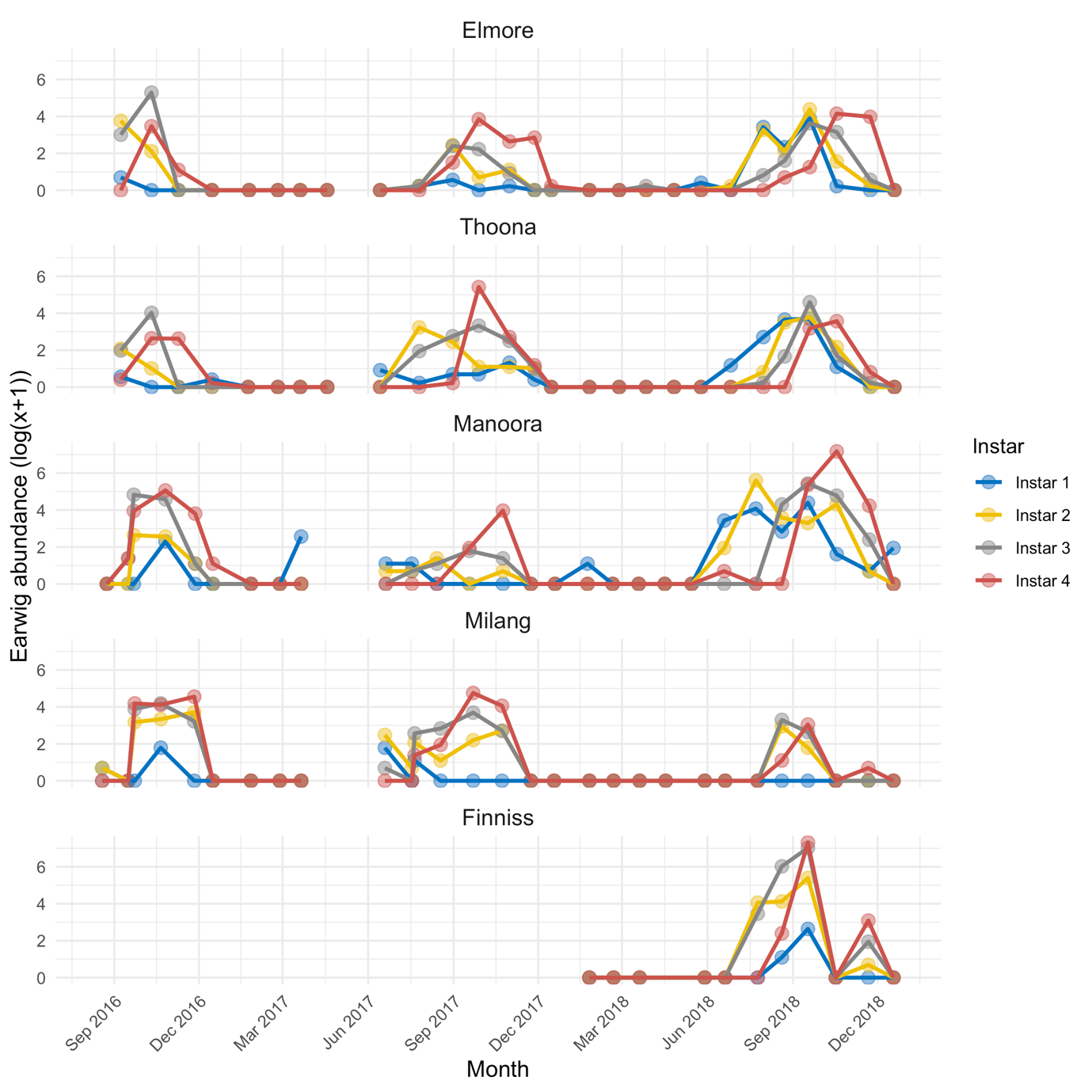


**Suppl. Fig. 3** Log mean abundance of the four *Forficula auricularia* juvenile instar stages collected from the three trap types at each field site. Total individuals for each site are: Elmore = 3190, Thoona = 3162, Manoora = 3582, Milang = 1459, Finniss = 3661.


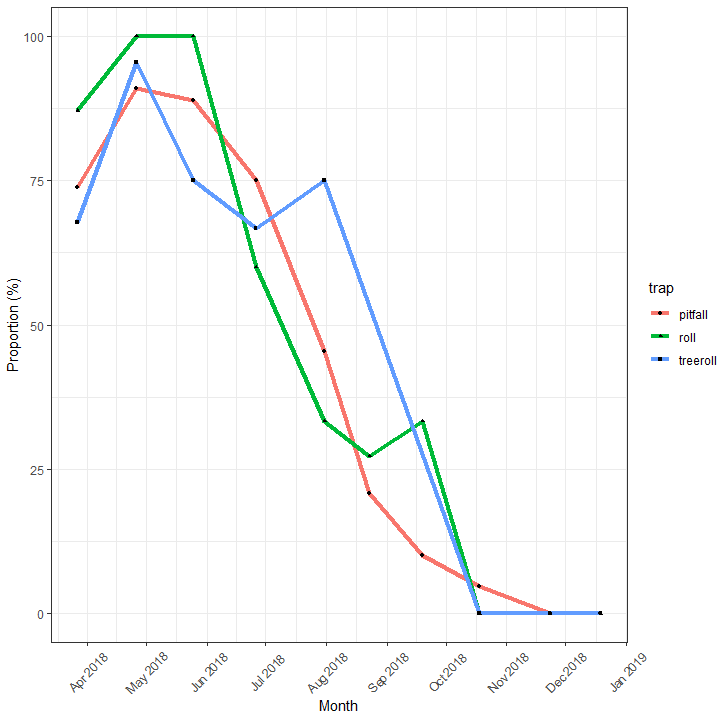


**Suppl. Fig. 4** Proportion of female *Forficula auricularia* that contained one or more eggs. Sites pooled, separated by trap type. Total number of females dissected to check for eggs = 676.
